# Differences between Hepatic and Cerebral Regional Tissue Oxygen Saturation at the Onset of Intradialytic Hypotension

**DOI:** 10.3390/jcm12154904

**Published:** 2023-07-26

**Authors:** Shohei Kaneko, Susumu Ookawara, Kiyonori Ito, Saori Minato, Yuko Mutsuyoshi, Yuichiro Ueda, Keiji Hirai, Yoshiyuki Morishita

**Affiliations:** Division of Nephrology, First Department of Integrated Medicine, Saitama Medical Center, Jichi Medical University, 1-847 Amanuma-cho, Omiya-ku, Saitama 330-8503, Saitama-ken, Japan; shohei.sasurai@gmail.com (S.K.); kiyonori.ito@gmail.com (K.I.); saorim0404@gmail.com (S.M.); mutsuyoshiyuko@yahoo.co.jp (Y.M.); mini_bouz_butterfly@yahoo.co.jp (Y.U.); keijihirai@kfy.biglobe.ne.jp (K.H.); ymori@jichi.ac.jp (Y.M.)

**Keywords:** cerebral oxygenation, hepatic oxygenation, intradialytic hypotension, splanchnic circulation, ultrafiltration rate

## Abstract

Background: Intradialytic hypotension (IDH) is a critical pathological condition associated with all-cause mortality in patients undergoing hemodialysis (HD). However, few studies have investigated IDH-related changes in hepatic and cerebral regional tissue oxygen saturation (rSO_2_). This study investigated IDH-induced changes in hepatic and cerebral rSO_2_. Methods: Hepatic and cerebral rSO_2_ during HD were measured using an INVOS 5100C oxygen saturation monitor, and their percentage (%) changes during the development of IDH were analyzed. Ninety-one patients undergoing HD were investigated, including twenty with IDH. Results: In patients with IDH, % changes in hepatic and cerebral rSO_2_ decreased at the onset of IDH. Additionally, the % change in hepatic rSO_2_ was significantly larger than that in cerebral rSO_2_ (*p* < 0.001). In patients without IDH, no significant differences were found between the % changes in hepatic and cerebral rSO_2_ at the time of the lowest systolic blood pressure during HD. Multivariable linear regression analysis showed that the difference between the % changes in cerebral and hepatic rSO_2_ was significantly associated with the development of IDH (*p* < 0.001) and the ultrafiltration rate (*p* = 0.010). Conclusions: Hepatic and cerebral rSO_2_ significantly decreased during the development of IDH, and hepatic rSO_2_ was more significantly decreased than cerebral rSO_2_ at the onset of IDH.

## 1. Introduction

The management of blood pressure in patients undergoing hemodialysis (HD) is a very important topic for clinicians [1]. Furthermore, elevated blood pressure is a widely known cardiovascular risk factor in patients undergoing HD [2]. To achieve a target blood pressure, a combination of various antihypertensive drugs is administered [1]. On the other hand, the clinical importance of hypotension during HD is also receiving increasing attention [3,4]. Intradialytic hypotension (IDH) is defined as symptomatic hypotension during hemodialysis and is associated with cardiovascular diseases, cerebrovascular disease, vascular access thrombosis, and all-cause mortality in patients undergoing HD [3,4]. This means that clinicians treating patients undergoing HD should be aware of the unique blood-pressure variability of patients undergoing HD. Clinicians should also have an in-depth understanding of the mechanisms of blood-pressure variability and compensatory mechanisms.

In previous studies, IDH-induced organ damage has been explained by impaired organ perfusion [3,4]. However, unexpectedly, only a few studies have empirically examined IDH and organ perfusion. The major reason for the insufficient evidence regarding IDH and organ perfusion is the difficulty in measuring organ blood flow during HD. Organ blood-flow measurement using magnetic resonance imaging (MRI) or positron emission tomography (PET) is challenging to apply because continuous and dynamic changes in the patient’s condition are difficult to detect during HD in real time [5,6,7]. Additionally, catheter-based organ blood-flow measurements are difficult because of their high invasiveness [7]. Therefore, the relationship between IDH and organ blood flow is still hypothetical and represents a significant challenge for researchers.

The clinical importance of measuring the regional tissue oxygen saturation (rSO_2_) using near-infrared spectroscopy has recently gained great attention [8,9,10,11,12]. Measurement of the rSO_2_ is a real-time and non-invasive method for evaluating organ tissue oxygenation in various clinical settings [8,9,10,11,12]. The rSO_2_ is not a parameter that directly indicates organ blood flow, but it assesses the organ tissue oxygenation, which is calculated using the ratio of oxyhemoglobin and deoxyhemoglobin in the targeted tissue. However, the rSO_2_ reflects the organ blood flow, because it is significantly associated with the organ blood flow measured using MRI and PET [5,6]. Therefore, the relatively straightforward measurement of rSO_2_ enables us to assess organ blood flow in the patient’s condition during HD. We have previously focused on the hepatic and cerebral rSO_2_ in patients undergoing HD and reported the clinical importance of their changes in different clinical situations related to HD treatment [8,9,10,11]. Interestingly, hepatic and cerebral rSO_2_ appear to change asynchronously under various HD conditions (e.g., blood transfusion during HD, treatment of congestive heart failure with HD, and feeding during HD) [13,14,15]. For example, during HD with ultrafiltration for congestive heart failure, the hepatic rSO_2_ appears to recover faster than its cerebral counterpart [13]. Moreover, hepatic rSO_2_ appears to recover more than the cerebral rSO_2_ when patients with severe anemia who are undergoing HD receive blood transfusions [14]. Currently, only a few studies have focused on IDH-induced changes in organ rSO_2_ [9,12]. Moreover, no clinical research has concurrently detected IDH-induced changes in rSO_2_ in multiple organs. Therefore, we aimed to investigate IDH-induced changes in hepatic and cerebral rSO_2_, and to explore the importance of the difference between the two rSO_2_ changes.

## 2. Materials and Methods

### 2.1. Ethical Approval

This study was approved by the Review Board of the Saitama Medical Center, Jichi Medical University (RIN15-104 and RINS19-HEN007) and complied with the Declaration of Helsinki (revised in Tokyo in 2004).

### 2.2. Study Design and Participants

This prospective cross-sectional study was conducted at the dialysis center of the Saitama Medical Center, Jichi Medical University. Hepatic and cerebral rSO_2_ during HD were measured (once per patient) in the patients undergoing HD. Detailed information about the study’s protocol was provided to patients undergoing HD, and those who agreed to participate in this study and signed the written consent form were included. The inclusion criteria were as follows: (i) patients who agreed to participate in this study, and (ii) patients aged < 20 years undergoing HD. The following were the exclusion criteria: (i) <1 month after starting HD, (ii) patients with congestive heart failure and severe neurological disorders, (iii) patients who developed the lowest SBP within 1 h after HD initiation, (iv) patients who received intradialytic feeding, and (v) patients with a history of renal transplantation. Recruitment of the study’s participants was conducted between 1 August 2013 and 31 December 2019.

### 2.3. Collection of Clinical Information

Clinical information, excluding rSO_2_, was obtained from medical records. This included age, sex, anthropometric data (e.g., body mass index), comorbidities (e.g., cardiovascular and cerebrovascular diseases), HD vintage, medication (e.g., antihypertensive drugs and vasopressors), laboratory data (e.g., hemoglobin (Hb), sodium, and albumin), transcutaneous oxygen saturation, ultrafiltration rate (UFR), and blood pressure. In our institute, blood pressure was measured every 30 min during HD, and additional measurements were conducted when intradialytic symptoms developed. The HD vintage (years) is the length of time for which a patient has been on HD treatment since its introduction in the past. HD time (hours) is the amount of time for which a patient is treated in one treatment session. In this study, some value changes were analyzed as the percentage (%) change. The % changes were calculated as [(value at the point in time of minimum systolic blood pressure (SBP) − value at baseline)/value at baseline × 100]. In this study, cardiovascular diseases included coronary artery disease, arrhythmia, and systolic or diastolic cardiac failure. Cerebrovascular disease included history of stroke or cerebral hemorrhage and carotid artery stenosis. Antihypertensive drugs included calcium channel blockers, renin–angiotensin system inhibitors, alpha-adrenergic blocking agents, beta-adrenergic blocking agents, and diuretics. The type and dosage of antihypertensive drugs were determined as appropriate by the dialysis specialist according to the Dialysis Treatment Guidelines in Japan [16].

### 2.4. Measurement of rSO_2_

Hepatic and cerebral rSO_2_ data during HD were measured using the INVOS 5100C oxygen saturation monitor (Covidien Japan, Tokyo, Japan), whose detailed measurement mechanisms have been previously described [14]. Briefly, this measurement device is composed of a light-emitting diode that transmits two wavelengths (735 and 810 nm) of near-infrared light and two light detectors to measure oxygenated and deoxygenated Hb. The rSO_2_ is calculated based on the ratio of the signal strengths of the oxygenated Hb and the total Hb (oxygenated Hb + deoxygenated Hb) [17,18]. The light detector obtains two signals at two different depths: 30 and 40 mm, as superficial and deep tissues, respectively. By analyzing these differences in the signals, the rSO_2_ at 20–30 mm from the body’s surface can be measured [19,20]. This measurement is conducted once every 6 s and recorded automatically. Before HD, the patients rested for at least 10 min in a supine position to reduce the effect of postural changes on their rSO_2_ values. For the measurement of hepatic rSO_2_, the sensor device was attached to the right intercostal area above the liver after the correct position was confirmed through ultrasonography. In contrast, the sensor device to measure cerebral rSO_2_ was attached to the patient’s forehead. Cerebral and hepatic rSO_2_ levels were monitored thereafter from 5 min after HD initiation to the end of HD. To track cerebral and hepatic oxygenation, we evaluated the mean rSO_2_ every 5 min as a single rSO_2_ value during HD.

### 2.5. Definition of IDH

In accordance with previous guidelines, IDH was defined as (i) an intradialytic sudden decrease in SBP (>20 mmHg) with IDH-related symptoms such as muscle cramps and loss of consciousness [21], or (ii) an intradialytic sudden decrease in SBP (>30 mmHg) or mean blood pressure (MBP) (>10 mmHg) with IDH-related symptoms [16].

### 2.6. Statistical Analysis

Data are expressed as means ± standard deviations, or as medians and interquartile ranges. Statistical comparisons between the two groups were performed using Student’s *t*-test for variables with normal distribution, and with the Mann–Whitney U test for variables that did not show a normal distribution. The chi-squared test was used to assess the differences in clinical parameters between the two groups and was complemented by an adjusted residual analysis. Correlations between the difference in the % changes in hepatic and cerebral rSO_2_ and clinical parameters in patients undergoing HD were evaluated using Pearson’s correlation or Spearman’s rank correlation for data with normal and skewed distributions, respectively. Multivariate linear regression analysis was performed to extract independent factors of the differences in the two rSO_2_ changes. All analyses were performed using IBM SPSS Statistics for Windows, version 26.0 (IBM, Armonk, NY, USA). Any *p*-values < 0.05 were considered statistically significant.

## 3. Results

### 3.1. Patients’ Characteristics

Overall, 91 patients undergoing HD met this study’s criteria. Table 1 shows the clinical characteristics of the patients. The patients’ age was 70.0 (63.0–76.0) years, 69 (76%) patients were male, and their mean body mass index was 22.4 (19.9–25.0) kg/m^2^. Twenty (22%) patients developed IDH in this study, and all complained of muscle cramps as IDH-related symptoms. The values of hepatic and cerebral rSO_2_ at baseline were 55.8 ± 15.3% and 50.2 ± 10.1%, respectively. In contrast, the hepatic and cerebral rSO_2_ values at the lowest SBP were 53.8 ± 14.9% and 49.3 ± 9.7%, respectively. The % changes in hepatic and cerebral rSO_2_ were −2.8 ± 11.3% and −1.5 ± 6.4%, respectively. Furthermore, the % changes in SBP and MBP were −13.4 (−21.3–−7.6) % and −9.0 (−20.4–−5.0) %, respectively.

### 3.2. IDH-Related Changes in Hepatic and Cerebral rSO_2_

**Figure 1:**
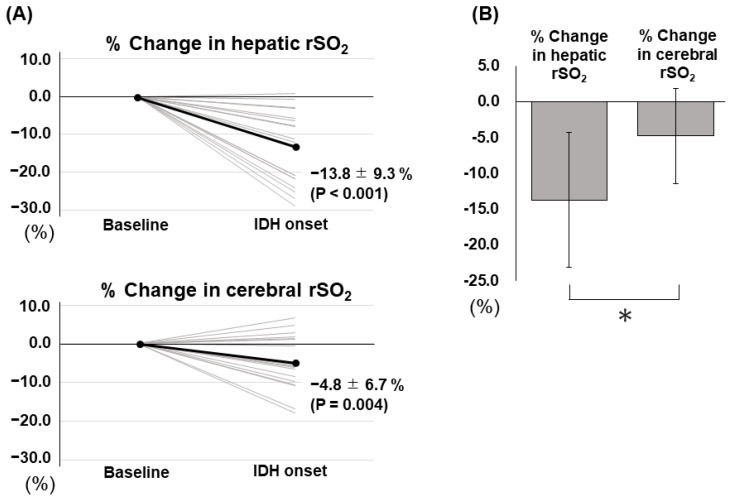
Percent changes in hepatic and cerebral rSO_2_ in the development of IDH (*n* = 20): (**A**) % changes in hepatic and cerebral rSO_2_ from baseline to the time of IDH onset. Grey line presented individual % change in hepatic and cerebral rSO_2_ and black line presented mean % changes in hepatic and cerebral rSO_2_. (**B**) Comparison between the % changes in hepatic rSO_2_ and in cerebral rSO_2_. Abbreviations: rSO_2_, regional tissue oxygen saturation; IDH, intradialytic hypotension. * *p* < 0.001.

Table 2 and Figure 1 show the IDH-related changes in hepatic and cerebral rSO_2_ in patients who developed IDH (*n* = 20). The % changes in hepatic and cerebral rSO_2_ significantly decreased to −13.8 ± 9.3% (*p* < 0.001) and −4.8 ± 6.7% (*p* = 0.004), respectively. Additionally, the % change in hepatic rSO_2_ was significantly larger than that in cerebral rSO_2_ (*p* < 0.001). The difference between the % change in cerebral rSO_2_ and that in hepatic rSO_2_ was 9.0 ± 9.9%. Clinical factors between the IDH (*n* = 20) and the non-IDH (*n* = 71) groups are described in Supplementary Table S1 online. In the IDH group, the % changes in hepatic (*p* <0.001) and cerebral rSO_2_ (*p* = 0.010) were significantly larger than those in the non-IDH group, and the difference between the two rSO_2_ changes was significantly higher in the IDH group than that in the non-IDH group (*p* < 0.001) (see Supplementary Figure S1 online). In patients without IDH, no significant differences were found between the % changes in hepatic and cerebral rSO_2_ (% changes in hepatic rSO_2_: 0.3 ± 9.8%; % changes in cerebral rSO_2_: −0.6 ± 6.1%; *p* = 0.426). Furthermore, the difference between the % changes in hepatic and cerebral rSO_2_ at the lowest SBP during HD was not significant in this study (−0.9 ± 9.8%, *p* = 0.426).

### 3.3. Factors Associated with the Difference between the Two rSO_2_ Changes

**Figure 2:**
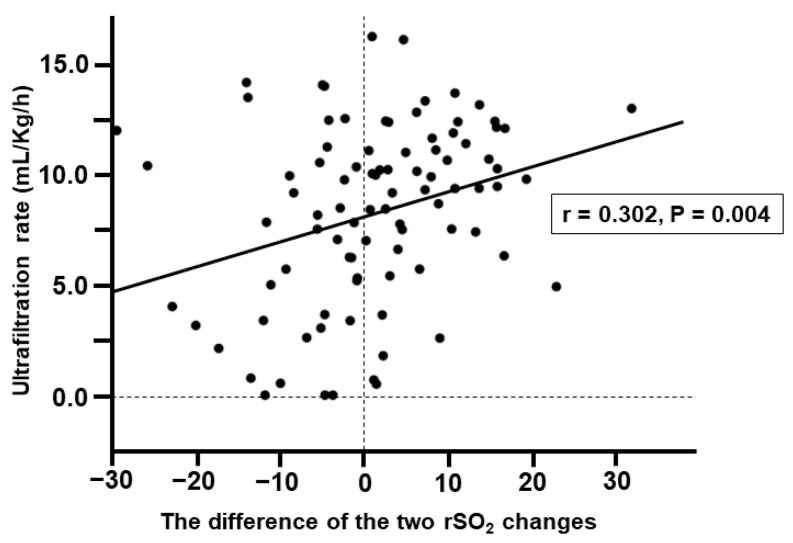
Association of the difference between the two rSO_2_ changes and the ultrafiltration rate. The difference between the two rSO_2_ changes was calculated using “% change in cerebral rSO_2_ − % change in hepatic rSO_2_”. The statistical association between the two is shown with a scatterplot. Abbreviations: rSO_2_, regional tissue oxygen saturation; r, correlation coefficient.

The clinical factors associated with the difference between the % changes in cerebral and hepatic rSO_2_ were investigated with univariate and multivariate analyses (Table 3). Univariate analysis showed that UFR (*p* = 0.004) and the development of IDH (*p* < 0.001) were significantly associated with the difference between the two rSO_2_ changes. In contrast, multivariable linear regression analysis using these factors as explanatory variables showed that the difference in the two rSO_2_ changes was significantly associated with UFR (*p* = 0.010) and the development of IDH (*p* < 0.001, Figure 2).

## 4. Discussion

In this study, we explored the IDH-induced changes in hepatic and cerebral rSO_2_. In summary, our findings showed that both hepatic and cerebral rSO_2_ significantly decreased upon the development of IDH, and that hepatic rSO_2_ decreased more markedly than cerebral rSO_2_ at the time of IDH onset. Additionally, the difference between the two rSO_2_ changes, calculated using “% change in cerebral rSO_2—_% change in hepatic rSO_2_”, was significantly associated with UFR and the development of IDH. These results can be considered important, as only a few previous studies have examined IDH-related changes in rSO_2_.

As mentioned in the Introduction section, rSO_2_ reflects the organ blood flow [5,6]. In this regard, hepatic and cerebral rSO_2_ indicate hepatosplanchnic and cerebral circulations, respectively [5,6,22]. Our study’s results suggested that hepatosplanchnic and cerebral circulation decreased with the onset of IDH, which was expected [3,4]. However, it was unexpected that hepatosplanchnic circulation decreased more significantly than cerebral circulation with the onset of IDH. Why is there a difference between the changes in hepatosplanchnic and cerebral circulations with the onset of IDH? The difference may be explained by the fact that each organ has different mechanisms for maintaining blood flow.

A patient’s body is constantly exposed to hypovolemic stress due to ultrafiltration during HD [3,4]. Therefore, the body performs various compensatory functions to maintain the central circulation under this condition, including activation of the sympathetic nervous system, arterial constriction, plasma refilling, and transfer of blood volume from organs to the central circulation [3,4]. These compensatory mechanisms allow the patient’s body to maintain the central circulation (i.e., cardiac output) and prevent impaired organ perfusion [3,4]. In these mechanisms, hepatosplanchnic circulation plays a particularly important role [3,4]. The hepatosplanchnic circulation is known to be the reservoir that supplies blood volume to the central circulation under hypovolemic stress; this mechanism is known as the De Jager–Krogh effect. Hepatosplanchnic circulation has a very large capillary bed. In the absence of arterial constriction (not under hypovolemic stress), the pressure in the capillary bed is increased, and blood is pooled due to the stasis. However, in the presence of arterial constriction (e.g., under hypovolemic stress), the pressure in the capillary bed is decreased, supplying blood to the central circulation [3,4]. Specifically, the central circulation is constantly maintained on the basis of the self-sacrificing effect of the hepatosplanchnic circulation, whereas the situation of cerebral circulation is greatly different. The brain has a mechanism to maintain cerebral blood flow even if systemic blood pressure fluctuates widely; this mechanism is known as cerebral autoregulation [23,24]. Cerebral blood flow is maintained by increasing cerebral vascular resistance when systemic blood pressure drops [23,24]. Classically, cerebral blood flow has been considered to be maintained at an MBP of 60−150 mmHg [23,24]. This range appears to be slightly different in patients undergoing HD; however, a recent study reported that the lower MBP limit (i.e., not inducing cerebral ischemia) is 74 mmHg [12]. At least, cerebral circulation appears to be protected under hypovolemic stress. When IDH develops due to triggers such as myocardial ischemia, hepatosplanchnic and cerebral circulation are negatively affected. In this case, hepatosplanchnic circulation would be greatly affected by its nature (i.e., self-sacrificing effect), whereas cerebral circulation would be minimally affected. This difference would have been indicated as the difference between hepatic and cerebral rSO_2_ in this study. This study evaluated rSO_2_, and not organ blood flow directly. However, we believe that there is no inconsistency with respect to previous reports.

This study was cross-sectional and cannot prove the causal relationships among parameters. However, it appears certain that the increased difference between the two rSO_2_ changes is an “unfavorable sign” associated with the development of IDH. Interestingly, the difference between the two rSO_2_ changes is also correlated with UFR. For clinicians, the optimization of UFR is a very important subject of discussion [25]. A high UFR (for example, >13 mL/h/kg) is associated not only with the development of IDH, but also with patient mortality [25,26,27,28]. Therefore, the optimal UFR should be customized for each patient, since it is influenced by the patient’s body size and disease background (particularly cardiac diseases) [25]. This result highlights the importance of optimizing the UFR to prevent circulatory failure (e.g., IDH development). This clinical message may appear obvious to many clinicians. However, most evidence on UFR optimization is based on observational studies. Therefore, we explained the importance of optimizing the UFR in this study using the rSO_2_ changes, which we measured.

This study has some limitations. First, there is a scope for further investigation regarding the relationship between rSO_2_ and organ blood flow. However, rSO_2_ remains the most promising method of assessing organ blood flow during HD at this time, despite these concerns. Second, this study was cross-sectional. Specifically, we only found correlations between parameters; therefore, it is difficult to draw any causal conclusions. Furthermore, analysis of real-time changes during HD may provide more insight into the causal relationship between organ rSO_2_ and IDH. Particularly, predicting the development of IDH using rSO_2_ changes would be an interesting research topic in the future. Third, the rSO_2_ values may have been influenced by various dialysis conditions, such as the dialysis setting and various physiological factors [9,10,11,13,14,15]. Fourth, the sample size in this study was small, and organ rSO_2_ was measured only once per patient. Regarding our future research prospects, we would like to increase the sample size and perform multiple rSO_2_ measurements in the same patient to further explore the relationship between IDH and rSO_2_.

## 5. Conclusions

Hepatic and cerebral rSO_2_ significantly decreased upon the development of IDH, and hepatic rSO_2_ was more significantly decreased than cerebral rSO_2_ at the time of IDH onset. Multivariable linear regression analysis showed that the difference between the % changes in cerebral and hepatic rSO_2_ was significantly associated with the development of IDH and the UFR. Therefore, larger-scale studies, including factor analysis, should be conducted to clarify this issue. Moreover, further large-scale studies are also required to accumulate data on rSO_2_ changes during the development of IDH.

## Figures and Tables

**Table 1:** Patients’ characteristics (*n* = 91).

Age (years)	70.0 (63.0–76.0)
Male sex, *n* (%)	69 (76)
Body mass index (kg/m^2^)	22.4 (19.9–25.0)
HD vintage (years)	0.6 (0.1–6.2)
Cardiovascular diseases, *n* (%)	35 (38)
Cerebrovascular diseases, *n* (%)	15 (16)
Administration of antihypertensive drugs, *n* (%)	84 (92)
Administration of vasopressor before HD, *n* (%)	11 (12)
Hemoglobin (g/dL)	9.8 ± 1.6
Sodium (mEq/L)	137 ± 4
Albumin (g/dL)	3.1 (2.8–3.6)
O_2_ saturation (%)	96.0 (93.7–97.2)
Ultrafiltration rate (mL/kg/h)	9.3 (5.4–11.4)
Development of IDH, *n* (%)	20 (22)
Hepatic rSO_2_ before HD (%)	55.8 ± 15.3
Hepatic rSO_2_ at the lowest SBP (%)	53.8 ± 14.9
% Change in hepatic rSO_2_ (%)	−2.8 ± 11.3
Cerebral rSO_2_ before HD (%)	50.2 ± 10.1
Cerebral rSO_2_ at the lowest SBP (%)	49.3 ± 9.7
% Change in cerebral rSO_2_ (%)	−1.5 ± 6.4
SBP before HD (mmHg)	147 ± 24
Lowest SBP (mmHg)	122 ± 24
% Change in SBP (%)	−13.4 (−21.3–−7.6)
SBP after HD (mmHg)	146 ± 24
MBP before HD (mmHg)	100 ± 17
MBP at the lowest SBP (mmHg)	86 ± 18
% Change in MBP (%)	−9.0 (−20.4–−5.0)
MBP after HD (mmHg)	99 ± 15

Categorical data are presented as numbers (%); continuous data are presented as the mean ± standard deviation or median and interquartile range. Abbreviations: HD, hemodialysis; IDH, intradialytic hypotension; rSO_2_, regional tissue oxygen saturation; SBP, systolic blood pressure; MBP, mean blood pressure.

**Table 2:** IDH-related changes in hepatic and cerebral rSO_2_ (*n* = 20).

Variables	
% Change in hepatic rSO_2_ (%)	−13.8 ± 9.3
% Change in cerebral rSO_2_ (%)	−4.8 ± 6.7
Difference between the two rSO_2_ changes (%)	9.0 ± 9.9
% Change in SBP (%)	−23.8 (−32.5–−10.6)
% Change in MBP (%)	−24.9 (−37.6–−20.6)

Abbreviations: rSO_2_, regional tissue oxygen saturation; SBP, systolic blood pressure; MBP, mean blood pressure. The difference between the two rSO_2_ changes was calculated using “% change in cerebral rSO_2_ − % change in hepatic rSO_2_”.

**Table 3:** Factors associated with the difference between the two rSO_2_ changes (*n* = 91).

	Simple Linear Regression		Multivariable Linear Regression	
Variables	r	*p*	Standardized Coefficient	*p*
Age	−0.045 ^#^	0.670		
Male sex	−0.148	0.161		
Body mass index	0.014 ^#^	0.896		
HD vintage	0.138 ^#^	0.191		
Cardiovascular diseases	0.044	0.682		
Cerebrovascular diseases	−0.122	0.251		
Administration of hypertensive drugs	0.136	0.199		
Administration of vasopressors before HD	0.143	0.178		
Hemoglobin	0.013	0.900		
Sodium	−0.118	0.265		
Albumin	0.008 ^#^	0.938		
O_2_ saturation	0.203 ^#^	0.054		
Ultrafiltration rate	0.302 ^#^	0.004 *	0.250	0.010 *
Development of IDH	0.389	<0.001 *	0.356	<0.001 *

* Statistically significant; ^#^ indicates Spearman’s rank correlation for skewed distribution of data. The difference between the two rSO_2_ changes was calculated using “% change in cerebral rSO_2_ − % change in hepatic rSO_2_”. Abbreviations: HD, hemodialysis; IDH, intradialytic hypotension; rSO_2_, regional tissue oxygen saturation.

## Data Availability

All data analyzed during this study are available within the paper.

## Supplementary Materials

The following supporting information can be downloaded at: https://www.mdpi.com/article/10.3390/jcm12154904/s1. Figure S1. Comparison of the IDH (*n* = 20) and non-IDH (*n* = 71) groups. Table S1. Comparison of the clinical factors between the IDH and non-IDH groups (*n* = 91).
