# Supplementary material for: Differences between Hepatic and Cerebral Regional Tissue Oxygen Saturation at the Onset of Intradialytic Hypotension
Source: J Clin Med. 2023 Jul 26;12(15):4904. doi: 10.3390/jcm12154904 (PMC10419901; doi:10.3390/jcm12154904)
Supplement: Supplementary file 1 [file jcm-12-04904-s001.zip › Supplementary Figure S1.pdf]

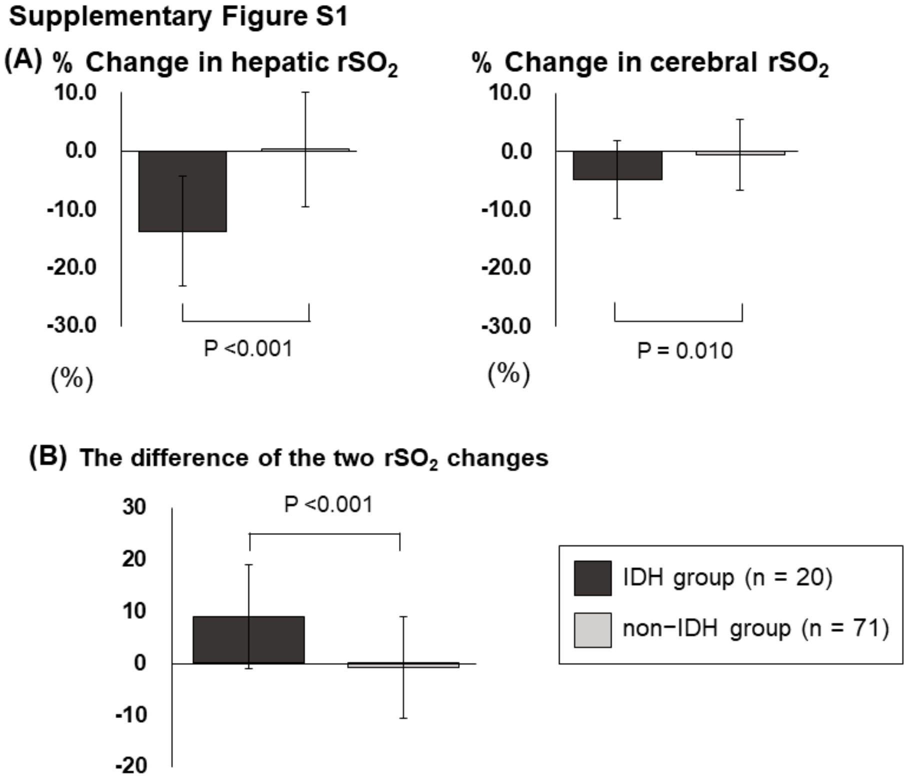

Supplementary Figure S1. Comparison of the IDH (n = 20) and non-IDH (n = 71) groups. (A) % changes in hepatic and cerebral rSO_2 in the IDH and non-IDH groups. (B) Difference between the two rSO2 changes in the IDH and non-IDH groups. Difference in the two rSO2 changes was calculated using "% change in cerebral rSO_2 - % change in hepatic rSO_2."

Abbreviations: rSO_2, regional tissue oxygen saturation; IDH, intradialytic hypotension.
