# Supplementary material for: Differences between Hepatic and Cerebral Regional Tissue Oxygen Saturation at the Onset of Intradialytic Hypotension
Source: J Clin Med. 2023 Jul 26;12(15):4904. doi: 10.3390/jcm12154904 (PMC10419901; doi:10.3390/jcm12154904)
Supplement: Supplementary file 1 [file jcm-12-04904-s001.zip › Supplementary Table S1.pdf]

Supplementary Table S1. Comparison of the clinical factors between the IDH and non-IDH groups (n = 91)

Variables	IDH group (n = 20)	Non−IDH group (n = 71)	P-value
Age (years)	70.5 (64.0 – 73.0)	71.0 (62.0 – 76.0)	0.533
Male sex, *n* (%)	14 (70)	55 (77)	0.491
Body mass index (kg/m^2)	22.4 (19.8 – 25.2)	22.9 (21.1 – 24.5)	0.605
Cardiovascular diseases, *n* (%)	9 (45)	26 (37)	0.496
Cerebrovascular diseases, *n* (%)	2 (10)	13 (18)	0.587
HD vintage (years)	4.3 ± 6.1	4.5 ± 7.3	0.900
Administration of hypertensive drugs, *n* (%)	18 (90)	66 (93)	0.971
Administration of vasopressor before HD, *n* (%)	6 (30)	5 (7)	0.017
Hemoglobin (g/dL)	9.8 ± 1.8	9.8 ± 1.5	0.999
Sodium (mEq/L)	137 ± 4	137 ± 3	0.097
Albumin (g/dL)	3.1 (2.7 – 3.5)	3.1 (2.8 – 3.6)	0.535
O_2 saturation (%)	96.7 (95.6 – 97.5)	95.9 (92.7 – 97.2)	0.062
Ultrafiltration rate (mL/kg/h)	10.1 (8.0 – 11.1)	8.4 (5.2 – 11.9)	0.224
% Change in hepatic rSO_2 (%)	-13.8 ± 9.3	0.3 ± 9.8	< 0.001
% Change in cerebral rSO_2 (%)	-4.8 ± 6.7	-0.6 ± 6.1	0.010
Difference between the two rSO_2 changes (%)	9.0 ± 9.9	-0.9 ± 9.8	< 0.001
SBP before HD (mmHg)	143 ± 22	148 ± 25	0.431
Lowest SBP (mmHg)	100 ± 21	128 ± 21	< 0.001
SBP after HD (mmHg)	138 ± 19	148 ± 24	0.091
MBP before HD (mmHg	96 ± 16	101 ± 16	0.211
MBP at the lowest SBP (mmHg)	72 ± 18	90 ± 16	< 0.001
MBP after HD (mmHg)	91 ± 14	101 ± 15	0.015
% Change in SBP (%)	-23.8 (-32.5 – -10.6)	-7.6 (-16.3 – -4.2)	< 0.001
% Change in MBP (%)	-24.9 (-37.6 – -20.6)	-11.6 (-17.1 – -6.5)	< 0.001

Abbreviations: SD, standard deviation; HD, hemodialysis; rSO_2, regional tissue oxygen saturation; SBP, systolic blood pressure; MBP, mean blood pressure. The deference in the two rSO_2 changes was calculated using "% change in cerebral rSO_2 - % change in hepatic rSO_2".
